# Sharing is healing: The power of storytelling to transform mental health in the field of neglected tropical diseases

**DOI:** 10.1371/journal.pmen.0000400

**Published:** 2025-08-12

**Authors:** Anil Fastenau, Rhea Brüggemann, Nervana Ibrahim, Abdul Salam, Lucy Mckane, Ali Murtaza, Fabian Schlumberger, Inés Egino, Daniel Wainstock, Srilekha Penna, Ngozi Ekeke, Daniel Gonzalo Eslava Albarracin, Hamideh Ebrahimi, Alexandra Asboeck, Matthew Willis

**Affiliations:** 1 Marie Adelaide Leprosy Center, Karachi, Pakistan; 2 German Leprosy and Tuberculosis Relief Association (DAHW), Wuerzburg, Germany; 3 Department of Global Health, Institute of Public Health and Nursing Research, University of Bremen, Bremen, Germany; 4 Heidelberg Institute of Global Health, University of Heidelberg, Heidelberg, Germany; 5 Department of International Public Health, Liverpool School of Tropical Medicine, Liverpool, United Kingdom; 6 Anesvad Foundation, Bilbao, Spain; 7 Pontifical Catholic University of Rio de Janeiro, Rio de Janeiro, Brazil; 8 German Leprosy and Tuberculosis Relief Association (GLRA) India, New Delhi, India; 9 RedAid Nigeria, Enugu, Nigeria; 10 La Escuela de Ciencias de la Salud, Universidad Nacional Abierta y a Distancia (UNAD), Bogota, Colombia; 11 Lahore School of Nursing (LSN), University of Lahore, Lahore, Pakistan; PLOS: Public Library of Science, UNITED KINGDOM OF GREAT BRITAIN AND NORTHERN IRELAND

## Introduction

Neglected tropical diseases (NTDs) affect more than a billion people globally, disproportionately impacting the most marginalized and socioeconomically vulnerable communities [1,2]. NTDs such as buruli ulcer, cutaneous leishmaniasis, leprosy and noma are not only illnesses of the body but also impact the mental and social well-being of those affected [3]. While effective medical treatments exist, people affected by these neglected diseases continue to suffer as a result of stigma, social exclusion, and a range of other invisible mental health burdens [4]. In many low-resource settings where NTDs are still prevalent, their psychosocial impacts such as internalized stigma, shame, anxiety, and depression can be more disabling than the disease itself [4]. Addressing these harms requires more than medicine; it demands social innovation. One such innovation is participatory storytelling [5,6]. When people affected by NTDs are empowered to tell their own stories through art, music, poetry, acting, singing, dancing, film, or just spoken words, they do more than narrate suffering: they reclaim identity, affirm agency, and challenge dominant narratives of fear and exclusion [7]. People affected by NTDs heal by sharing their stories in their own ways of expression. This innovative approach, piloted by the German Leprosy and TB Relief Association (GLRA) and partners in Colombia, Ethiopia, Nigeria, Pakistan, India, and other countries, demonstrates how storytelling can become a contact intervention that both reduces stigma and improves mental wellbeing of people affected by NTDs.

## The hidden psychosocial burden of NTDs

**Fig 1:**
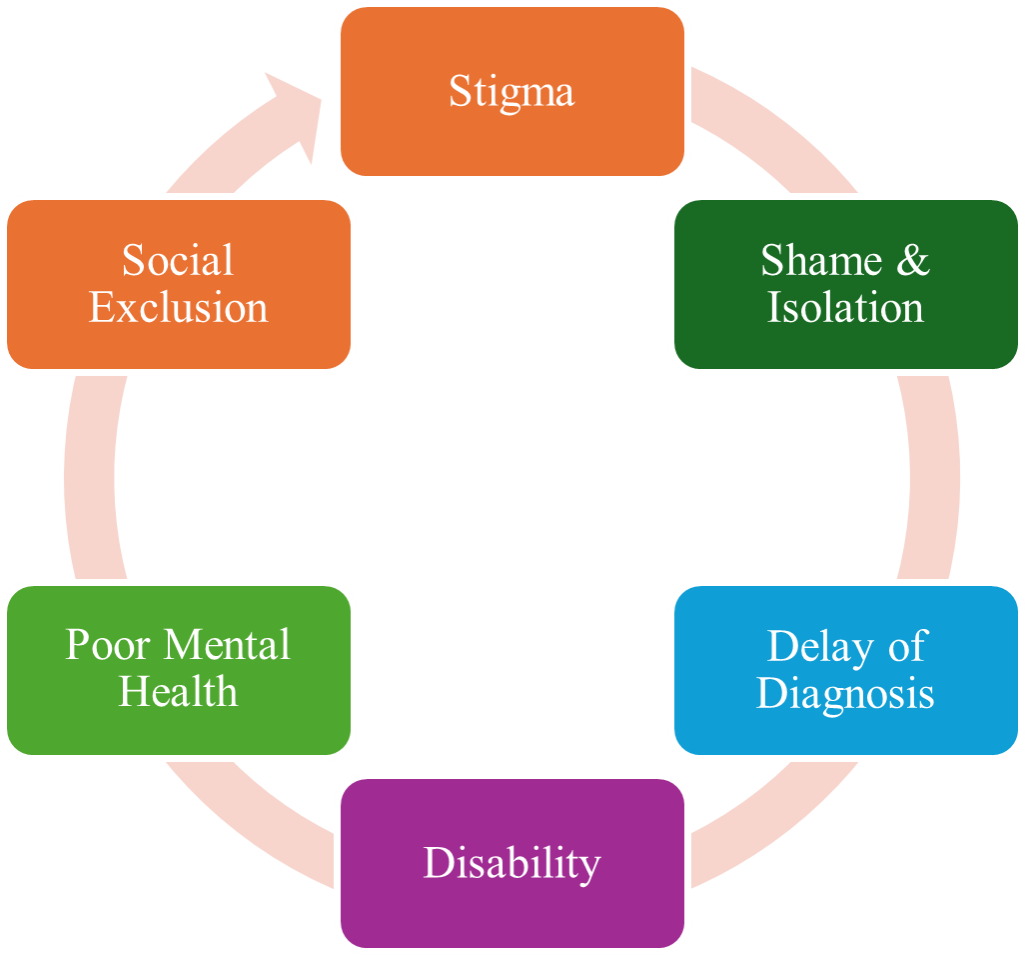
Vicious cycle of stigma.

NTD-related stigma forms a vicious cycle: fear of disease leads to social exclusion, which in turn delays health-seeking behavior, increasing the risk of visible disability further, thus reinforcing stigma [4]. This cycle also results in poor mental health outcomes, including internalized shame, reduced self-worth, and isolation [8]. Mental distress is often compounded by structural barriers, such as loss of employment, diminished educational opportunities, and lack of access to healthcare [8]. Fig 1 below illustrates the vicious cycle of stigma. Therefore, addressing stigma is crucial for the success of the fight against NTDs [9], as it directly affects early diagnosis, treatment, and social reintegration of people affected by NTDs.

Yet stigma reduction interventions and mental health support remain grossly underfunded and underprioritized in global NTD strategies [10,11]. For example, although the WHO’s Global Leprosy Strategy (2021–2030) includes goals of “zero stigma and discrimination,” operationalizing this ambition remains a challenge. Only a few interventions meaningfully address the psychosocial needs of people affected by NTDs [10,12], and even fewer include their voices as agents of change.

## Storytelling as a contact intervention

Drawing on both theoretical and empirical foundations, participatory storytelling emerges as a transformative public health tool [7,13]. Grounded in empowerment theory and contact intervention models, interactive storytelling facilitates direct and indirect contact between stigmatized groups and the broader community, thereby reducing prejudice and fostering empathy [7,14].

A recent initiative in Pakistan, led by German Leprosy and TB Relief Association and Marie Adelaide Leprosy Centre (MALC) brought together 25 individuals affected by leprosy for a one-year project called “Storytelling Game Changer” [15]. The initiative began with a two-week participatory storytelling workshop, where 25 individuals with lived experience of leprosy were trained through acting, singing, music, dance, photography, and narrative writing, followed by monthly meetings throughout the one-year project cycle [15]. The workshop served as the foundation for the project, equipping participants with skills and support to craft and share their personal testimonies. Following the initial workshop, regular storytelling sessions were facilitated by the organizers, including several follow-up sessions to measure changes in participants’ mental health and wellbeing. The stories of the participants were shared via film screenings and community events, reaching both healthcare professionals and the public [15]. The response was overwhelmingly positive, participants described the experience as healing, empowering, and deeply validating. As one participant reflected, “For the first time, I felt seen.”

This aligns with similar initiatives conducted in India by GLRA, where participatory storytelling methods were used to reduce internalized stigma and improve mental health [16]. In India, baseline and endline assessments using tools like PHQ-9 (Patient Health Questionnaire-9) and 5-QSI-AP (5-Question Stigma Indicator for Affected Persons) scales indicate improvements in psychological wellbeing and reductions in internalized stigma. The results of these research studies from both India and Pakistan are currently being finalized and will soon be submitted to scientific journals for publication, aiming to document the strong evidence supporting storytelling as a powerful and innovative tool for empowerment, stigma reduction and mental health promotion.

## Why storytelling works

There are several reasons why storytelling is particularly effective in this context:

1. **Restoring Voice and Agency**: NTDs often rob individuals of autonomy, dignity, and self-expression. Storytelling re-establishes a sense of control which is central to psychological empowerment [17].
2. **Humanizing the Experience**: Rather than being viewed as passive recipients of help, persons affected by NTDs become relatable, dynamic individuals with hopes, fears, and aspirations [18].
3. **Collective Healing**: Sharing stories within a peer group fosters solidarity and shared resilience. This collective narrative-building can transform shame into strength [6,13].
4. **Culturally Rooted Expression**: In many societies, including Colombia, Pakistan and India, storytelling is a valued cultural practice. Integrating this familiar medium into public health interventions ensures cultural resonance and community ownership.

## From local innovation to global movement

The success of these pilot initiatives has led to a vision for global scale-up. Through partnerships with local International Federation of Anti-Leprosy Associations (ILEP) members and organizations of persons affected by NTDs, storytelling-based stigma reduction interventions are being implemented in Brazil, Colombia, Ethiopia, and Nigeria. Importantly, this model is not a “one-size-fits-all” solution. Each context requires tailoring, ensuring that the stories resonate locally while contributing to global solidarity. Beyond its psychosocial impact, this participatory approach also builds sustainable capacity. Participants are trained as peer facilitators, storytellers and video-makers, enabling them to carry the torch forward in their communities. The use of simple, low-cost technology such as mobile phones makes this a scalable and replicable intervention.

## Policy implications

To fully leverage the potential of storytelling as a mental health, stigma-reduction and empowerment tool in NTDs, several policy recommendations emerge:

- **Integrate Mental Health in NTD Programs**: Mental health support, including community-based psychosocial interventions, must be an essential component of national NTD strategies [11].
- **Fund Participatory Approaches**: Donors and implementing agencies should prioritize participatory, arts-based approaches not as optional add-ons, but as core components of stigma and mental health programming.
- **Promote Lived Experience Leadership**: Persons affected by NTDs must be involved not only as participants but as co-designers, facilitators, and evaluators of interventions.
- **Strengthen Monitoring and Evaluation**: Use of validated tools should be embedded into program design to track mental health outcomes and guide adaptive learning.

## Toward a new narrative

**Fig 2:**
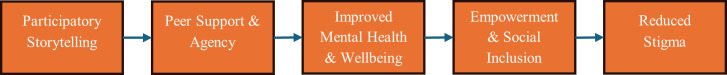
Transformative pathway of participatory storytelling.

“Sharing is healing,” as articulated by several participants, underscores the transformative potential of participatory storytelling in the context of neglected tropical diseases (NTDs). As illustrated in Fig 2, participatory storytelling fosters peer support and agency, which contributes to improved mental health and wellbeing. This process further enhances empowerment and social inclusion, ultimately leading to reduced stigma within communities.

These narratives extend beyond accounts of suffering; they represent testimonies of resilience, resistance, recovery, renewal and success. By investing in participatory storytelling, NTD programs achieve more than stigma reduction; they contribute to shifting the narrative surrounding NTDs from one of exclusion to one centered on empowerment and social justice.

The mental health needs of persons affected by NTDs have historically been under-recognized within health systems and programming. It is now imperative to listen not only with empathy but to act systematically, embedding participatory storytelling approaches into integrated NTD and mental health strategies to address psychosocial burdens while dismantling the structures of stigma and exclusion.
